# Endogenous Hormone Regulation During Key Developmental Stages of *Pinus koraiensis* Siebold & Zucc. Ovules

**DOI:** 10.3390/plants14050637

**Published:** 2025-02-20

**Authors:** Xueqing Liu, Xiaoqian Yu, Ling Yang

**Affiliations:** 1 College of Forestry, Beijing Forestry University, Beijing 100091, China; 2 State Key Laboratory of Tree Genetics and Breeding, Northeast Forestry University, Harbin 150040, China; lxq19846151691@163.com (X.L.); yxq384811@163.com (X.Y.)

**Keywords:** Korean pine, cone, development, fertilization process, anatomical structure, endogenous hormones

## Abstract

In this study, the morphological and anatomical characteristics of the growth of the internal ovules and the dynamic changes in the content of endogenous hormones during the development of Korean pine (*Pinus koraiensis* Siebold & Zucc.) cones were investigated in detail and their interrelationships determined. In addition, morphological examinations, paraffin section, analysis and enzyme immunoassays were performed to observe the growth and development as well as the fertilization stages of the ovules of *P. koraiensis* from July of the pollination year to June of the following year. From July of the pollination year to May of the next year, the increase in the content of indoleacetic acid (IAA) and gibberellin and a decrease in the content of abscisic acid (ABA) in the pollination year correlated with the division of the gametophyte free nuclei. It was observed that the levels of IAA, ABA, zeatin riboside (ZR) and isopentenyl adenosine (IPA) initially decreased and then increased during overwintering, which was interpreted as a symptom of adaptation of *P. koraiensis* ovules to low temperatures. At the end of overwintering, the increase in IPA, ZR and ABA levels was associated with the development of the female gametophyte. The week before fertilization was identified as the stage of oocyte division, in which growth-promoting hormones dominate. During the week of fertilization, the increase in the level of growth-inhibiting hormones correlated with fertilization. After fertilization, the increase in the level of growth-promoting hormones also correlated with early embryonic development. The levels of endogenous hormones were observed to change dynamically with the development of *P. koraiensis* oocytes, indicating their important role. The results of this study provide the morphological and anatomical basis for related studies on the development of the ovarian strobilus in gymnosperms.

## 1. Introduction

The sexual reproduction of plants includes the differentiation of flower buds, pollination, fertilization and embryogenesis. Studies on plant sexual reproduction have gained importance in plant science due to the long-term collection of research data and the development of molecular biology techniques [1]. The embryos of conifers are the result of the fertilization of an egg cell. The reproductive mechanisms of this insemination process differ from those of the double fertilization observed in angiosperms [2,3]. Compared to angiosperms, the study of gymnosperms is incomplete due to the long development cycle and difficulty of observation.

Generally, pinecones mature in the next year after pollination [4]. When the cones are mature, the ovulate scales develop into seed scales, while the ovules in the cones develop into seeds. As the site of sexual reproduction in plants [5], the formation and development of the ovule occupies a central position in the life history of alternating diploid and haploid generations in plants [6]. *Pinus contorta* female gametophytes overwinter during the free-nuclear stage, and fertilization occurs in mid-June after overwintering. Embryos and seeds mature in early August [7]. The fertilization of *Pinus tabulaeformis* occurs in May of the second year after pollination. During the development of the ovule, the first nucellar tapetum layer begins to appear at the same time as the mother cells of the megaspore [8]. Next, the macrospore is due to meiosis of the archesporial cell; then, karyokineses of the haploid nuclei bring about the stage of the free nuclei gametophyte. At this stage, the tapetum cells near the gametophyte contain many small liposomes. The next step is alveolation and the determination of the archegonial initials. Concurrently with the above, the macrospore cell wall expands; this wall is a common outer wall of the whole female gametophyte [9]. Macrospore mother cell development is accompanied not only by significant morphological changes [10] but also by dynamic changes in its endogenous hormones and nutrient content. Mutations that lead to a change in these contents often result in female infertility [11,12,13].

Endogenous hormones regulate the reproductive growth process of plants. The explanation of hormone level changes can provide a theoretical basis for in-depth research on generative development and its underlying mechanism. Recently, studies on the changes in endogenous hormones during plant sexual reproduction have primarily focused on angiosperms, and studies on endogenous hormones in gymnosperms are limited. Different tree species undergo different changes in endogenous hormone levels during fertilization. In *Pinus taeda* the abscisic acid (ABA) content in the fertilized female gametophyte continuously decreases [14]. Furthermore, a study on *Pseudotsuga menziesii* reported that the contents of indole-3-acetyl-l-aspartate and ABA-glucose ester in a conjugated form are higher than those of indoleacetic acid (IAA) and ABA during fertilization [15]. During the development of *Pinus koraiensis* embryos at different stages, the contents of indoleacetic acid (IAA), zeatin riboside (ZR), abscisic acid (ABA), and gibberellins (GAs) were analyzed, revealing that IAA, ABA, and ZR play crucial regulatory roles in this process; however, GAs do not have a significant impact on this developmental process [16]. Although this conclusion enriches our understanding on the sexual reproduction of *P. koraiensis*, the important period from pollination to fertilization remains unelucidated.

*P. koraiensis*, the main group tree in the deciduous forests of *P. koraiensis*, is an extremely vigorous tree species, with excellent wood quality and nutritious “nuts” in China and East Asia [17]. To ensure successful fertilization and cone maturation, the development of the ovule of *P. koraiensis* takes about 12 months [18]. However, there is currently no detailed explanation of the internal structure and dynamic changes in hormone content at this stage. Therefore, the observation of the female gametophyte and the determination of the chemical substances during the growth process of *P. koraiensis* can provide a basis for the research of the physiological process of reproductive growth of *P. koraiensis* and the underlying molecular mechanism. In addition, they can provide a theoretical basis for crossbreeding, artificial pollination and improved seed selection of *P. koraiensis*, as well as technical support for the introduction of accurate technologies to control seed formation in *P. koraiensis*. In the present study, the main developmental stages were determined by analyzing the morphology and paraffin sections of the ovules of *P. koraiensis*. In addition, the dynamic changes of five endogenous hormones, namely IAA, ZR, isopentenyladenosine (IPA), GA_3_ and ABA, were analyzed. Finally, the determination of endogenous hormone levels was combined with anatomical observations to understand the relationship between structural changes and endogenous hormone levels and to reveal the regulatory role of endogenous hormones in the development of the ovule of *P. koraiensis*.

## 2. Results

### 2.1. Morphology of P. koraiensis Cones

**Figure 1:**
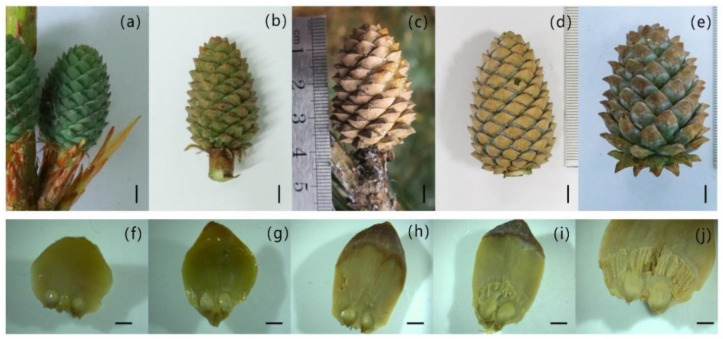
Morphological changes in the cones and ovules of *P. koraiensis* at different stages of development. Note: (**a**–**e**) The photos of post-pollination cones were taken in, respectively, July, August, and November of the year of post-pollination and in March and May of the second year of post-pollination; bar = 0.5 cm. (**f**–**j**) The photos of seed scales with ovules in the above periods; bar = 500 μm.

The cones of *P. koraiensis* were green in mid and late July of the pollination year; in mid and late August the color intensity decreased and the seed scales turned yellow (Figure 1a,b). In mid-November, all seed scales turned yellow-brown, with distinct color changes. During this period, the size of the cones increased, with the transverse diameter increasing from 1.47 cm to 2.67 cm (an average monthly increase of 0.3 cm); in addition, the longitudinal dimension increased from 2.8 cm to 3.7 cm, with an average monthly increase of 0.23 cm. From November of the pollination year to March of the following year, the cones were in the overwintering stage. Although the morphology of the *P. koraiensis* cones did not change obviously, the entire cone was pale yellow (Figure 1c,d). The cone size increased slowly, with the diameter increasing from 2.67 cm to 2.87 cm (an average monthly increase of only 0.05 cm) and the longitudinal dimension increasing from 3.7 cm to 3.93 cm (an average monthly increase of only 0.06 cm). From March to May of the following year, the cone entered a vigorous growth stage. The seed scales turned green. At the same time, the degree of opening of the seed scales increased. In addition, the size of the cone increased rapidly, with the diameter increasing from 2.87 cm to 4.12 cm (an average monthly increase of 0.63 cm) and the longitudinal dimension increasing from 3.93 cm to 5.6 cm (an average monthly increase of 0.84 cm) (Figure 1e).

**Figure 2:**
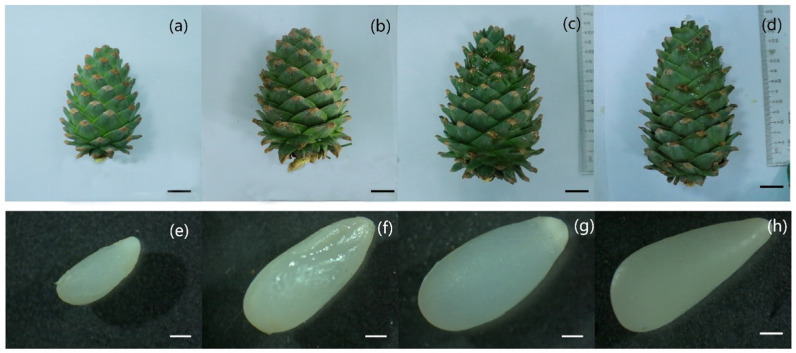
Morphological changes in cones and ovules of *P. koraiensis* during fertilization. Note: (**a**–**d**) The cones were photographed on 8, 15, 22, and 30 June in the second year after fertilization; bars = 2 cm. (**e**–**h**) Photos of ovules or developing seeds at the same time points, respectively; bars = 500 μm.

In June of the next year after pollination, the ovule was in the pre- and post-fertilization stage. The color of the seed scale changed from light green to dark green, with mucus oozing from the surface. At this stage, the diameter of the cone increased from 7.0 cm to 9.5 cm and the longitudinal dimension increased from 9.5 cm to 14.5 cm, indicating a steady increase. At this stage, the ovule changed from a translucent to a yellowish opaque. The final shape resembled that of mature seeds. In addition, the diameter of the ovule increased from 0.95 mm to 1.93 mm and the longitudinal dimension increased from 2.4 mm to 4.4 mm, especially from early June to mid-June (Figure 2).

### 2.2. Anatomical Observation of the Ovule of P. koraiensis

**Figure 3:**
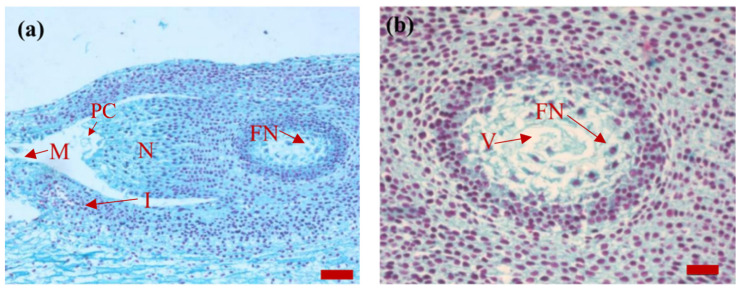
Images of the sagittal section of the *P. koraiensis* ovule on 15 July of the year of post-pollination. Note: In (**a**), bar = 100 μm. Image (**b**) is an enlarged image of image (**a**) (the same below), bar = 50 μm. M, micropyle; N, nucellus; PC, pollen chamber; I, integument; FN, free nucleus; V, vacuole.

*P. koraiensis* was pollinated in mid-June to late June of the flowering year. During pollination, two ovules were formed at the base of the seed scale. On 15 July, when the ovules were dissected, about 18 free nuclei (in a single section) were found in the developing female gametophyte. The nuclei were stained red and the cytoplasm was stained green; furthermore, the nuclei were deeply stained and the cytoplasm was dense. A vacuole was present in the free nuclei stage. The free nuclei were distributed fairly evenly around the vacuole. The nucellar tapetum was arranged in the form of concentric circles. Among them, 4–5 layers of the cells were closely arranged in the inner layer. The volume of the cells and nuclei was slightly larger than that of other nucellar cells, the nuclei were round and the cytoplasm was dense. The peripheral 3–5 layers of cells were loosely arranged, had oval nuclei and a slight coloration of the cytoplasm. The number of nucellar cells near the micropyle was lower, with only 2–3 layers and a relatively loose arrangement. However, the number of nucellar cells near the micropyle was relatively large, with about 4–5 layers (Figure 3).

**Figure 4:**
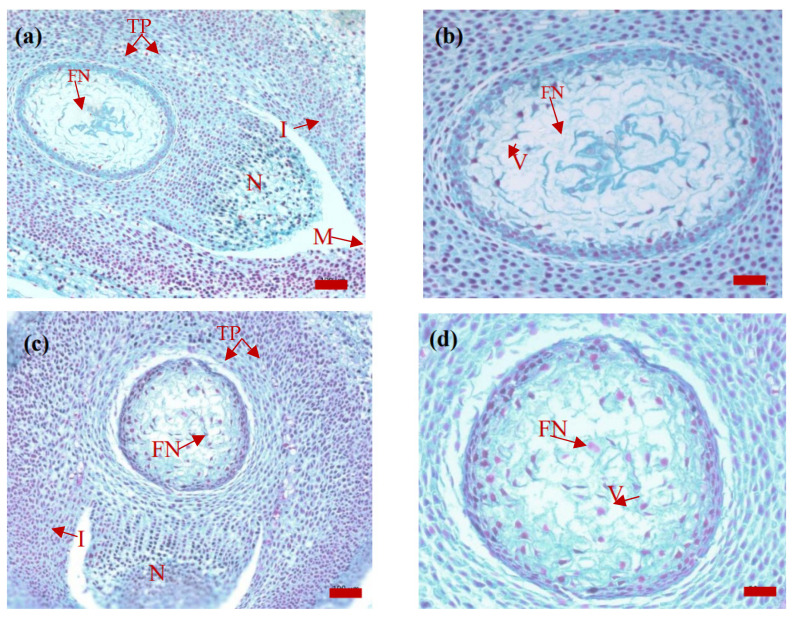
Images of *P. koraiensis the* ovule section on 15 August (**a**,**b**) and 15 December (**c**,**d**) of the year of pollination. Note: (**a**,**c**), bar = 100 μm; (**b**,**d**), bar = 50 μm. M, micropyle; N, nucellus; I, integument; FN, free nucleus; V, vacuole; TP, tapetum.

The free nuclei continued to divide, with approximately 26 nuclei on 15 August (Figure 4a) and 38 nuclei (in a single section) on 15 December (Figure 4c). The shapes of the free nuclei and cytoplasm were irregular. In addition, the color of the dividing free nuclei was light, but the cytoplasm was particularly dense. As the free nuclei divided, the number of free nuclei increased.

The tapetum cells were still arranged in two layers; 4–5 cell layers near the female gametophyte were densely arranged, the nucleus was oval, the color was light, and the cytoplasm was thick. Outside the tapetum nucellar, the 3–4 cells were loosely arranged, with oblong nuclei and a light color to the cytoplasm. There was a gap between the inner and outer layers of the nucellar tapetum.

**Figure 5:**
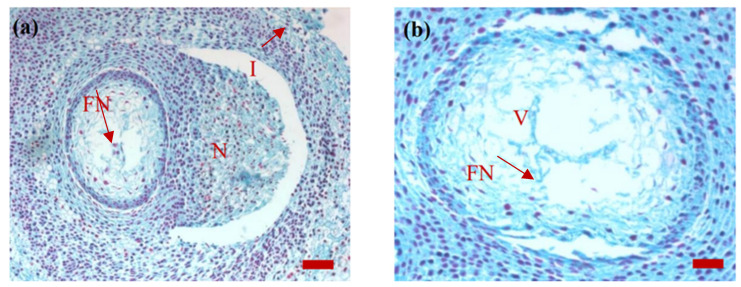
Images of the section of the ovule of *P. koraiensis* on 15 March of the year after pollination. Note: (**a**), bar = 100 μm; (**b**), bar = 50 μm. N, nucellus; I, integument; FN, free nucleus; V, vacuole.

In March of the year after pollination, the number of free nuclei did not increase compared to November of the previous year. At this time, the volume of free nuclei was larger, and the cytoplasm was thicker than before overwintering. However, there were no obvious changes in the tapetum cells (Figure 5).

**Figure 6:**
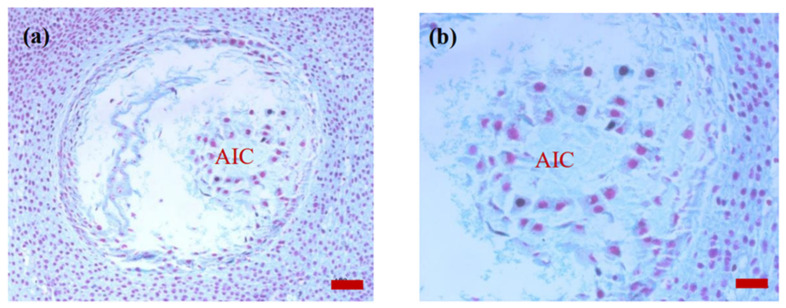
Images of the sections of the ovule of *P. koraiensis* on 15 April of the second year of pollination. Note: (**a**), bar = 100 μm; (**b**), bar = 50 μm. AIC, archegonial initial cell.

On 15 April of the next year after pollination (Figure 6a), the cells were clearly divided at the proximal micropylar end of the female gametophyte, differing morphologically from the surrounding cells. They formed archegonial initials that were neatly arranged in a circular pattern.

**Figure 7:**
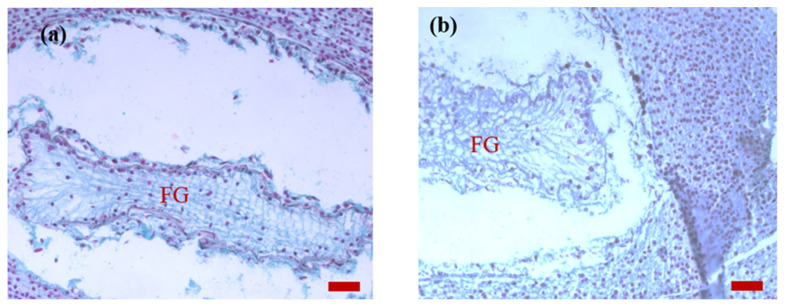
Images of the ovule sections of *P. koraiensis* on 15 May of the second year after pollination. Note: (**a**), bar = 100 μm; (**b**), bar = 50 μm. FG, female gametophyte.

On 15 May of the next year after pollination (Figure 7a), the cellular female gametophyte formed, which had an irregular lattice shape overall.

**Figure 8:**
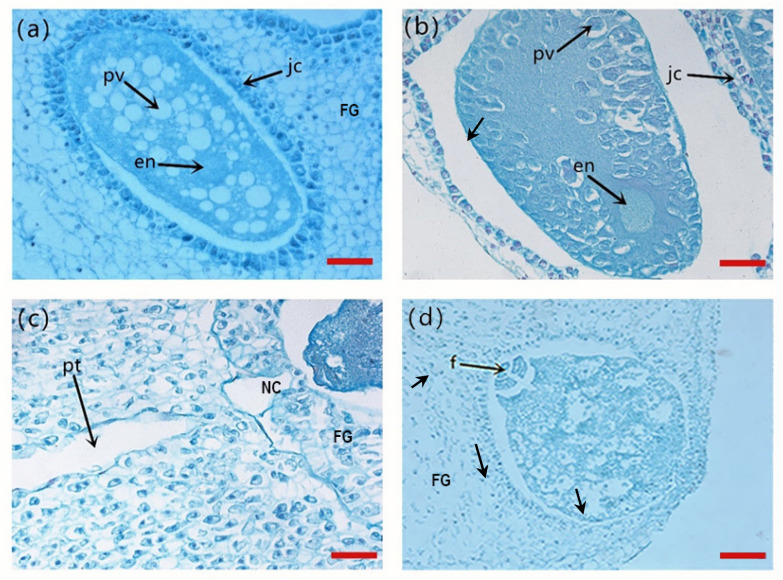
Longitudinal section of the ovule before and after the fertilization process of *P. koraiensis*. Note: (**a**–**c**), bar = 5 μm; (**d**), bar = 10 μm; pv, protein vacuole; jc, jacket cells; en, egg nucleus; pt, pollen tube; NC, neck cells; FG, female gametophyte; f, eight-cell embryo.

On 8 June, the year after pollination, the archegonia initials underwent a series of divisions to form egg cells. The egg cells were surrounded by jacket cells. The egg cells contained many protein vesicles (Figure 8a). The nucleus of the egg cells was located at the micropylar end of the female gametophyte and was granular, and no pollen tubes were observed near the archegonia. On 15 June of the following year after pollination, the protein vesicles disappeared and disintegrated, the cytoplasm became denser, and nucleus enlargement was observed (Figure 8b). On 24 June of the next year after pollination, a pollen tube apex was observed at the micropylar end of the female gametophyte, which was interpreted as the fertilization stage beginning (Figure 8c). On 30 June, an eight-cell embryo appeared in some ovules (Figure 8d). These results suggest that 8 to 15 June is the week before fertilization, 15 to 22 June is the week of fertilization, and 22 to 30 June is the week after fertilization.

### 2.3. Dynamic Changes in Endogenous Hormone Contents in the Ovules of P. koraiensis

#### 2.3.1. Changes in Endogenous Hormone Content During Ovule Development

**Figure 9:**
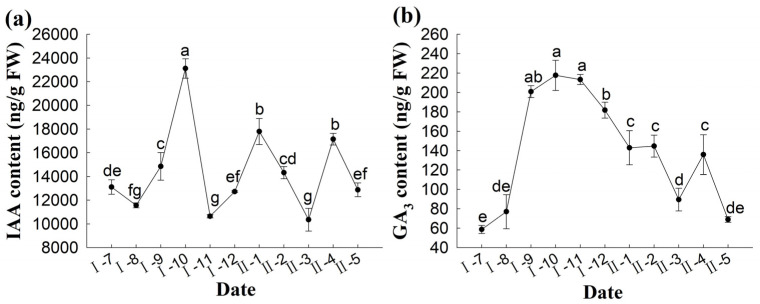
Changes in IAA and GA_3_ content during the development of the ovule of *P. koraiensis*. Note: (**a**) IAA content; (**b**) GA_3_ content. lowercase letters in the same sample indicate significant differences (*p* < 0.05). I—the year of pollination; II—the second year of pollination.

July to October of the pollination year was the period of cell divisions in the female gametophyte. The changes in IAA content showed an upward trend and peaked in October (23,117.39 ng·g^−1^ FW); therefore, we hypothesized that IAA plays an important role in the mitotic period of the female gametophyte. During overwintering, the IAA content in the ovule of *P. koraiensis* showed a dynamic trend, first decreasing and then increasing. After November, the average temperature decreased to <0 °C, the free nuclei stopped dividing, the ovule entered the overwintering stage, and the IAA content decreased to 10,658 ng·g^−1^ FW, which facilitated the adaptation of the ovule of *P. koraiensis* to the low-temperature environment (*p* < 0.05), after which the IAA content increased again (Figure 9a).

The GA_3_ content in the ovule of *P. koraiensis* initially increased and then decreased. During the mitotic period of the female gametophyte, i.e., from July to October of the pollination year, the GA_3_ content increased significantly (*p* < 0.05) and peaked in October (217.5827 ng·g^−1^ FW), indicating that the higher GA_3_ content was related to the growth and development of ovules after pollination. After November of the pollination year, when the temperature dropped to <0 °C, the GA_3_ content showed a significant downward trend (*p* < 0.05). These results indicate that the growth rate of the ovulated strobilus of *P. koraiensis* slowed down in a low-temperature environment and was related to low GA_3_ content (Figure 9b).

**Figure 10:**
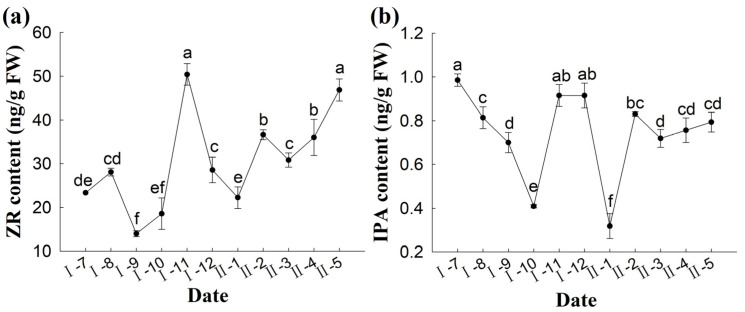
Changes in IPA and ZR content during the development of the ovule of *P. koraiensis*. Note: (**a**) ZR content; (**b**) IPA content. Different lowercase letters in the same sample indicate significant differences (*p* < 0.05). I—the year of pollination; II—the second year of pollination.

Measurement of the content of free ZR and bound IPA in the ovules of *P. koraiensis* revealed that ZR is the major cytokinin associated with ovule division and development (Figure 10a). The dynamic change trends of the contents of both were consistent. However, the relative content of IPA was low and can be neglected compared to that of ZR (Figure 10b). From pollination to overwintering, both ZR and IPA levels showed a decreasing trend. However, in November of the pollination year, the ZR content increased rapidly, reaching a peak of 50.4 ng·g^−1^ FW, before rapidly decreasing again. These results indicate that the cytokinin content decreases when the temperature drops. During overwintering, the cytokinin content of the ovule remained low that the ovule did not divide after adaptation to low temperatures and exhibited slower physiological activity until the end of overwintering. After overwintering, the free nuclei continued to divide, and the cytokinin content in the ovule of *P. koraiensis* increased significantly from March to May of the following year (*p* < 0.05).

**Figure 11:**
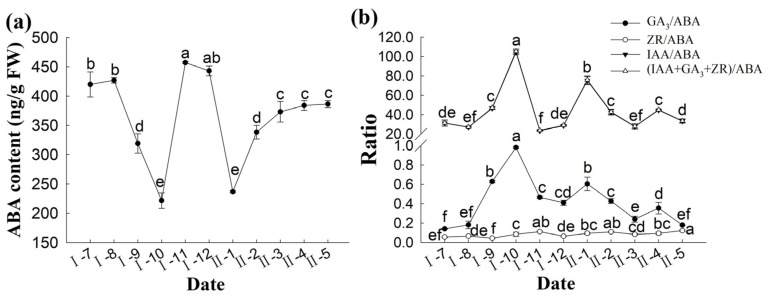
Changes in ABA content (**a**) and relative ratio during the development of the ovule of *P. koraiensis* (**b**). Note: different lowercase letters in the same sample indicate significant differences (*p* < 0.05). I—the year of pollination; II—the second year of pollination.

From July to October of the pollination year, the ABA content in the ovule of *P. koraiensis* decreased significantly (*p* < 0.05), indicating that the decrease in ABA content correlates with the development of the ovule. From March to May of the following year after pollination, the ABA content showed an insignificant upward trend (Figure 11a).

Plant growth and development are promoted by various plant hormones, which mutually restrict and promote each other. ZR, IAA and GA_3_ are growth-promoting hormones, while ABA is a growth-inhibiting hormone. As the IPA/ABA ratio was low, it was difficult to compare; therefore, no comparison was made. The levels of ZR and GA_3_ were significantly lower than those of IAA, the predominant growth-promoting hormone. The ratio of (IAA + ZR + GA_3_)/ABA was determined from the ratio of IAA/ABA and showed the same changing trend (Figure 11b). Although the ratios of IAA/ABA and GA_3_/ABA were quite different, they showed the same trend, with both showing a significant increase after pollination (*p* < 0.05). After the ratios peaked in October of the pollination year, they declined rapidly, with no significant changes in November and December. The ratios reached a second peak in January of the following year after pollination and then declined month by month until March of the following year; thereafter, the ratios increased and then declined in April of the following year. Changes in the ZR/ABA ratio were relatively stable, showing a general upward trend from pollination until November of the same year. They then decreased from November to December and remained relatively stable after an increase in January of the following year, with a significant increase in May of the following year.

#### 2.3.2. Changes in Endogenous Hormone Content During Egg Cell Fertilization

**Figure 12:**
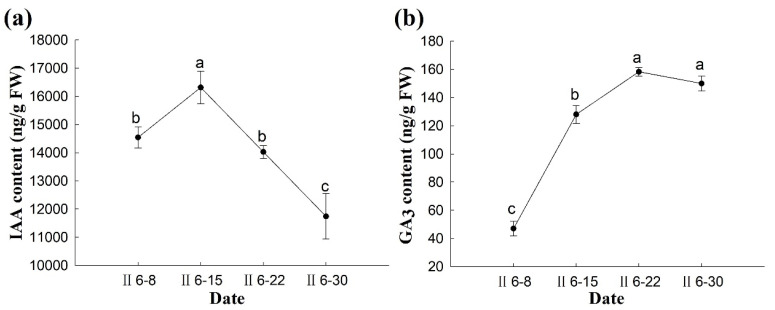
Changes in IAA and GA_3_ content during fertilization of the ovules of *P. koraiensis*. Note: (**a**) IAA content; (**b**) GA_3_ content. Different lowercase letters in the same sample indicate significant differences (*p* < 0.05); II is the second year of pollination.

In the week before fertilization, the IAA content increased significantly and peaked on 15 June (~16,316 ng·g^−1^ FW). The IAA content in the early stage of the zygotic embryos fell to the minimum value (11,740 ng·g^−1^ FW) one week after the week of fertilization (Figure 12a).

Figure 12b shows that the GA_3_ content increased significantly from 1 week before fertilization to the end of the fertilization week (*p* < 0.05). After fertilization, no significant difference in GA_3_ content was observed after fertilization; however, the change was relatively stable, suggesting that the GA_3_ content was not closely associated with fertilization of *P. koraiensis* (Figure 12b).

**Figure 13:**
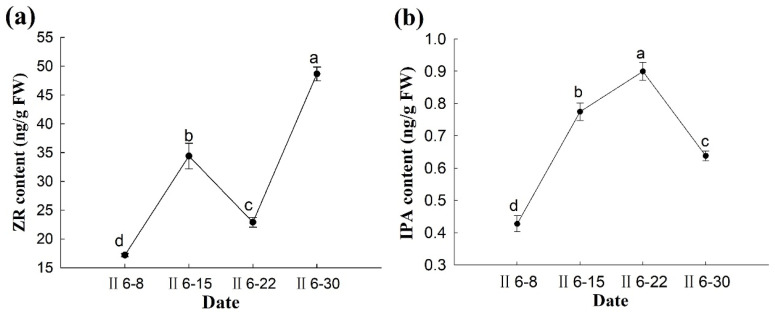
Changes in IPA and ZR content during the fertilization of the ovules of *P. koraiensis*. Note: (**a**) ZR content; (**b**) IPA content. Different lowercase letters in the same sample indicate significant differences (*p* < 0.05). II—the second year of pollination.

In the week before fertilization, the levels of ZR and IPA showed an upward trend. In the week of fertilization, the IPA content continued to increase and reached its maximum (~0.8996 ng·g^−1^ FW); in contrast, the ZR content briefly decreased to 22.893 ng g^−1^ FW. After fertilization, the IPA content decreased significantly (*p* < 0.05), while the ZR content increased significantly and reached its maximum (48.65667 ng·g^−1^ FW) (Figure 13).

**Figure 14:**
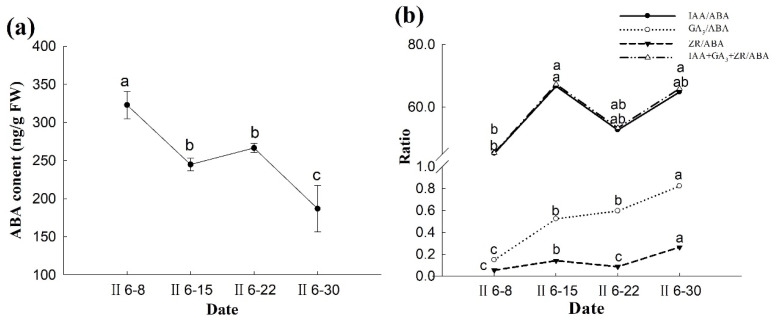
Changes in ABA content and relative ratios during the fertilization of the ovules of *P. koraiensis*. Note: (**a**) ABA content; (**b**) ratios. Different lowercase letters in the same sample indicate significant differences (*p* < 0.05). II—the second year of pollination.

The ABA content decreased in the week before fertilization but increased significantly in the week of fertilization. After fertilization, the ABA content decreased to the lowest value at the early stage of zygotic embryo division (~186.8667 ng·g^−1^ FW) (Figure 14a).

During fertilization, IAA was still the predominant growth-promoting hormone. The ratio of (IAA + ZR + GA_3_)/ABA was determined from the ratio of IAA/ABA and showed the same changing trend. Although the relative levels of IAA/ABA and ZR/ABA were very different, they showed the same trend. The relative content increased before fertilization, decreased around fertilization, and continued to increase after fertilization. In addition, the ratio of GA_3_/ABA was similar to the absolute content of GA_3_. The GA_3_/ABA content increased one week before fertilization and one week after fertilization, peaking on 30 June (Figure 14b).

## 3. Discussion

### 3.1. Anatomical Observation of the Development of the Ovule of P. koraiensis

The growth period of the cones of *P. koraiensis* is about 14–15 months, from ovule formation to seed maturation, with a period of winter dormancy. The development pattern of *Pinus radiata* is basically the same as that of other pine trees, but there are differences in time. Fertilization occurs 15 months after pollination, and morphological embryo maturity is reached 5 months later [3]. *Pinus monticola* also goes dormant in mid-July after their first spring pollination and then develops again in the second April [2]. In the present study, we comprehensively observed and determined the morphology, anatomical development, and endogenous hormone content of *P. koraiensis* during the whole development process.

First, the developmental process of the gametophyte of *P. koraiensis* was observed using the paraffin section technique. In the development process, the following stages were recognized: free nuclear division, archegonia development and egg cell development. The morphological and internal structural changes of each stage were clarified [10]. In 1987, Jin Chunying confirmed for the first time that the zygote undergoes four mitoses and finally divides into 16 cells [19]. However, due to the fact that the experimental photos in the above study are black and white images, unclear observations will hinder the progress of future tests. Based on the results of previous studies, in the present study, we comprehensively reported the ovule development and the process before and after the fertilization of *P. koraiensis*. The results of this study show that the nuclear stage in female gametophyte development occurs between 15 July of the pollination year and 15 March of the second year after pollination. However, in contrast to the results of previous studies, archegonial initials were observed in the ovule on 15 April of the year after pollination. These archegonial cells were also observed in the ovule of *P*. *tabuliformis* [20]. At the same time, it was observed that the female gametophytes gradually formed immature archegonia in the ovule on 15 May, the year after pollination. In *P. contorta*, mature female gametophytes with two to four neck cells were observed in mid-June of the year after pollination [7]. Inclusions of different sizes were observed on 8 June; this is similar to the anatomy of *Pinus sylvestris* [18]. In addition, many jacket cells were observed around the egg cells. Similarly, protein vacuoles were also observed in twisted pine ovules in mid-June [7]. Protein vacuoles are rich in proteins containing arginine, and the protein staining reaction of protein vacuoles in pine plants reaches its peak before fertilization [9]. Protein inclusions are most abundant in mature egg cells; however, they begin to decrease after fertilization. The appearance and disappearance of protein inclusions are therefore related to fertilization. Protein inclusions can provide nourishment for the fertilization of egg cells. However, the above conclusion on the effects of protein inclusions on conifers should be further investigated.

### 3.2. Effect of Endogenous Hormone Content on Ovule Development

The content of IAA, the main growth-promoting hormone, in the female gametophyte increased after pollination, suggesting that the higher IAA content may ensure successful division at the free nuclear division stage after pollination. In contrast to *P. koraiensis*, the IAA content of *Dacrydium pectinatum* reached the highest value at the ovule formation stage and gradually decreased during development [21]. In addition, IAA can improve the cold resistance of plants [22]. In the present study, the IAA content in the ovules first increased and then decreased. This is consistent with the responses of IAA in *Brassia campestris* and *Lavandula angustifolia* in a low-temperature environment [23]. After the temperature decreases, the growth rate slows down, which may reduce the damage to plants by low temperatures [24]. However, the change trends of IAA and cytokinin (ZR and IPA) during overwintering were the opposite, suggesting that auxin and cytokinin may have antagonistic effects during overwintering; however, the mechanism of action remains unclear.

GA_3_ plays an important role in the vegetative and reproductive growth of conifers, mainly by promoting early maturity, early flowering, and seed growth and development [21]. In the present study, GA_3_ levels increased significantly after pollination and peaked in mid-October, the stage of rapid division of free nuclei. This was accompanied by an increase in morphological indices, including longitudinal dimensions and weight, suggesting that the high GA_3_ content was associated with the division of the free nuclei and expansion of the female gametophyte after pollination. After the GA_3_ content peaked in October, it showed a declining trend during overwintering, suggesting that stress may reduce GA_3_ content in the ovule and thereby inhibit ovule growth [25]. In addition, the measurement data of morphological indices showed that the transverse and longitudinal dimensions of the ovules did not increase significantly during the overwintering period until the end of overwintering.

Cytokinin plays an important role in cell division and protein synthesis [21]. It has a positive effect on cell division and the expansion of the female gametophyte as well as on the uptake of nutrients required for the development of the embryo in the proembryonic stage. In the present study, IAA and ZR showed the same trend. However, the peak value of IAA appeared in October, while that of ZR appeared in November, i.e., with a delay of 1 month. Nevertheless, the overall trend was the opposite. IAA and ZR are also growth-promoting hormones, but their mechanisms and modes of action may be different. IAA and ZR may have antagonistic effects, which may complement each other in their content and keep hormone levels stable in plants. Bound IPA and free ZR are the most common cytokinins for regulating assimilate transport [26]. ZR levels were low during overwintering, which reduced cell division and was beneficial for plant overwintering. After overwintering, the ZR content increased, seed dormancy ended and the division of the free nuclei of the female gametophyte continued.

The role of ABA in pollination and fertilization has always been controversial. Some researchers think that a decrease in ABA content during pollination and fertilization is beneficial for normal fruit formation; however, other studies have reported that pollination can significantly promote ABA synthesis [21], although some caution is required as the phenomena in angiosperms may not be comparable to those in gymnosperms. In the present study, we observed that the ABA content in the pollinated ovules showed a downward trend. This could be due to the free nucleus division of the ovules after pollination, as a lower ABA content can accelerate the division process of the free nuclei. From November of the pollination year to February of the following year, the temperature remained below zero, and the cells in the leaves began to freeze, resulting in a physiological lack of water in the cells and a decrease in water content. This phenomenon is similar to drought stress and leads to a response of ABA to a cold environment and a sudden increase in ABA content [27].

There is an interaction between the different signaling transduction networks of hormones that regulate plant growth and development through cooperation or antagonism [28]. Therefore, different hormones and hormonal balance can promote pollination and fertilization as well as early embryogenesis. Different developmental stages require different doses and interactions of endogenous hormones [21]. Although the absolute levels of IAA and GA_3_ showed different trends, the ratios of IAA/ABA and GA_3_/ABA showed similar trends. Both ratios peaked in October of the year of pollination and then declined rapidly. The relative levels of growth-promoting hormones could increase to the maximum value due to some stimulation before overwintering and then rapidly decrease after the average temperature dropped to <0 °C in November. We suppose that the lower relative level of growth-promoting hormones is beneficial for overwintering and that after a brief increase following adaptation, the level remains low again until the end of overwintering. This conclusion is consistent with the research results of *B. campestris* and *L. angustifolia* [23,24]. The increase in ABA synthesis and the decrease in IAA synthesis could be beneficial for resistance to low temperatures [29,30]. In April of the next year after pollination, the archegonia cells began to divide and develop and resumed vigorous growth. However, the ZR/ABA ratio was relatively low and remained relatively stable. This indicates that the relative levels of these two hormones have little effect on the growth and development of the ovules.

### 3.3. Effect of Endogenous Hormone Content on Ovule Process Before and After Fertilization

As can be seen in Figure 12a, the IAA content increased significantly 1 week before fertilization, presumably because a large amount of IAA was involved in the process of egg division, leading to an increase in IAA content and reaching the peak. This conclusion is consistent with that of *P. menziesii* and *Picea asperata*; in both plants, IAA is necessary to support the development and fertilization of the female gametophyte [15]. However, the IAA content decreased rapidly during fertilization, which could be due to the fact that the pollen tube stopped growing after reaching the archegonium. One week after fertilization, the fertilized egg continued to divide, with an increasing trend in IAA content, indicating that the cell division of the fertilized egg cell requires a large amount of IAA. Sandberg and Ernsten reported that the free IAA content in the seeds of *Pinus densiflora* is high after fertilization, which is the active period of the development of the embryo [31].

The GA_3_ content increased before and after fertilization, suggesting that high GA_3_ content is beneficial for female gametophyte growth, organ expansion, and successful fertilization [32].

In the case of cytokinin, the conclusion of the present study supports previous data; that is, a decrease in the cytokinin content around fertilization is beneficial for successful fertilization [33]. After fertilization, the ZR content increased significantly, indicating that the embryo requires a large amount of ZR. This is consistent with the research results for *P. menziesii*; i.e., cytokinin levels decrease during the week of fertilization and increase rapidly after fertilization [17]. During the development of the female gametophyte of *P. menziesii*, the detection of IPA showed that this cytokinin may be necessary for promoting protein synthesis in this stage; however, whether IPA can promote protein synthesis during the development of the female gametophyte of *P. koraiensis* remains to be determined.

The ABA content decreased one week before fertilization, presumably because the lower ABA content was advantageous for the elongation of the pollen tube to the archegonium [21]. During the week of fertilization, the ABA content increased slightly indicating that a relatively high ABA content may promote successful fertilization [34]. Furthermore, this suggests that ABA plays a role in promoting the fertilization of *P. koraiensis*; this finding is consistent with Zeng’s research results on the empty-bud phenomenon in *Castanea mollissima* [35]. After fertilization, the ABA content decreased to the lowest level at the early embryo stage, indicating that low ABA content can ensure successful division in this stage. Three weeks before and after fertilization, the overall ABA content showed a decreasing trend. This was consistent with the previous research conclusions for *P. menziesii*; that is, a relatively low ABA content is required during fertilization [17]. In the second week after fertilization, the ABA content rapidly increased. A high ABA content can therefore stimulate vegetative growth of the endosperm; it is also beneficial for nutrient supply to the endosperm during the growth and development of the embryo, thus ensuring the successful growth of the embryo cells.

The week before fertilization is the stage of pollen tube elongation and egg cell expansion. Therefore, the ratios of IAA/ABA, ZR/ABA, GA_3_/ABA, and (IAA + ZR + GA_3_)/ABA were elevated at this stage suggesting that growth-promoting hormones play a major role before fertilization. However, during fertilization, the ratios of IAA/ABA, ZR/ABA, and (IAA + ZR + GA_3_)/ABA decreased, indicating that the ratio of growth-promoting hormones to growth-inhibiting hormones is low during fertilization; this is beneficial for successful fertilization. At the same time, combined with the absolute content of ABA, higher ABA content is beneficial for fertilization, similar to the condition in *Ginkgo biloba* that produces higher ABA content and lower IAA content during fertilization [36]. After fertilization, the ratios of growth-promoting hormones to growth-inhibiting hormones showed an upward trend. A higher ratio may be beneficial for early embryo division and development; this is consistent with research results for *Mangifera indica* [37].

## 4. Materials and Methods

### 4.1. Experimental Materials

*P. koraiensis* cones were from family No. 425 (127°27′32″ E, 42°13′36″ N), with 10 rows and 30 columns, five districts, second district, Hongwei Seed Orchard, Baishan City, Jilin Province, China, which consists of excellent clonal grafting mother trees planted in 1989. The age of the regenerated plants in 2020 was 36 years. Pollination was free, and the material was collected from June of the pollination year (2020) to mid-May of the next year (2021) and on 8, 15, 22, and 30 June of the following year (2021) after pollination. In the Hongwei Seed Orchard, 15 cones were sampled, immediately placed in a container with a built-in ice pack, and sent to the laboratory. In the laboratory, the ovules were excised from the seed scales with tweezers and dissecting needles, and 20 of the removed ovules were taken and put into FAA fixing solution (90 mL of 70% alcohol + 5 mL of glacial acetic acid + 5 mL of formaldehyde). After pumping air, the sample solution was stored in a refrigerator at 4 °C for 4 days to prepare paraffin sections. A total of 30 ovules were frozen in liquid nitrogen and stored at −80 °C for the determination of endogenous hormone content.

### 4.2. Morphological and Anatomical Observations of the Cones

According to the above sampling period, the appearance and morphology of fresh cones in the key period of cone development of *P. koraiensis* were observed and recorded. Five cones per stage were randomly selected and photographed with a Canon EOS 200D DSLR camera (Canon, Shanghai, China) (18–55 mm ISTM). The widest and longest dimensions of the cone were measured.

Immediately after taking the photos, the ovules were peeled off from the cones. Then, 3–5 ovules with scales were removed and photographed with a stereomicroscope (Stemi 508, Carl Zeiss, Beijing, China). The ovules that were well fixed were selected for the classical paraffin sectioning method to make permanent slides, with slight modifications based on a specific test method described previously [38]. In the modified method, the dehydration and wax soaking times were extended. The specific method is 70% alcohol 2 h, 85% alcohol 1 h, 95% alcohol 1 h, 95% alcohol 1 h, 100% alcohol 1 h, 100% alcohol 1 h, 1/2 xylene and 1/2 ethanol 1 h, xylene 1 h, xylene 1 h, and paraffin wax 2 h (three times). Safranin O and Fast Green staining was performed, and images were taken and observed using a ZEISS Primostar 3 biological microscope (Leica) (Carl Zeiss, Beijing, China)). To avoid errors and ensure that the developmental state presented by ovules in each period was repeated more than five times in each period, the most complete state was selected for taking the photographs.

### 4.3. Determination of the Contents of Endogenous Hormones in Ovules

In this step, 1 g ovules stored in the refrigerator at −80 °C were taken for measurements. The content of endogenous hormones in the plant was determined using an enzyme-linked immunosorbent assay. The ELISA kit (96T) was provided by Shanghai Enzyme Linked Biotechnology Co., Ltd. (Shanghai, China). The contents of GA_3_, IAA, ZR, IPA, and ABA were measured using a microplate reader (Infinite F50, Tecan, Männedorf, Switzerland). Plant hormones were extracted and determined according to the manufacturer’s instructions. The experiment was repeated 3 times in each period [39].

### 4.4. Data Analysis

All experimental data were analyzed using Microsoft Excel 2016 (Microsoft Corporation) and SPSS19.0 (V19, SPSS Inc., Chicago, IL, USA), tested for significance using Duncan’s new complex polar difference method, and plotted using SigmaPlot (V15.0, SYSTAT). Three biological replicates were used for each treatment.

## 5. Conclusions

The developmental process of ovules in the macrosporophyll of *P. koraiensis* was observed on the basis of morphology and anatomy, and the content of endogenous hormones was measured. The corresponding relationship between morphology and anatomical development was established, and the dynamic change in hormone content was also determined. Our study shows that ovule development, fertilization, and embryo development are regulated by different kinds of endogenous hormones after pollination. After pollination in summer and before overwintering, the relative contents of IAA and GA_3_ in ovules increased gradually and the relative contents of ABA gradually decreased, which regulated the expansion of the outer morphology of the megaspore sphere and the division of free nuclei in the ovules. During overwintering, the absolute contents of IAA, ABA, ZR, and IPA in the ovule decreased, and the morphology of the ovulate strobilus and the number of free nuclei in the ovule stabilized. After overwintering, the increase in the absolute content of IPA, ZR, and ABA in the ovule led to an increase in the morphology of the ovular strobilus, an increase in the number of free nuclei in the ovule, and the development of female gametophytes. In the spring of the following year, the egg cell division one week before fertilization was regulated by the increase in the relative contents of endogenous hormones IAA, GA_3_, and ZR. During the fertilization week, the relative content of growth-inhibiting hormone (ABA) increased. After fertilization, the relative of growth-promoting hormone (ZR) increased again to promote the early development of the embryo.

The anatomical observation in this study provided a theoretical basis for accurate sampling, and the determination of endogenous hormone content provided a reference for the subsequent application of exogenous hormones to promote cone and embryonic development. Meanwhile, the results of this study also provided a basis for proteome and transcriptome analysis at the molecular level and for the investigation of the molecular regulatory mechanisms of ovule development in *P. koraiensis*.

## Data Availability

The datasets supporting the conclusions of this article are included within the article.
